# Supplementary material for: Psychological Profiles in Ulcerative Colitis and Crohn’s Disease: Distinct Emotional and Behavioral Patterns
Source: Biomedicines. 2025 Jul 10;13(7):1694. doi: 10.3390/biomedicines13071694 (PMC12292374; doi:10.3390/biomedicines13071694)
Supplement: Supplementary file 1 [file biomedicines-13-01694-s001.zip › biomedicines-3666857-supplementary.pdf]

Table S1. Description and descriptive statistics of the MMPI-2 scales included in the study. The table summarizes all administered scales and subscales of the Minnesota Multiphasic Personality Inventory-2 (MMPI-2), including: (1) Validity Scales; (2) Clinical Scales; (3) Clinical Subscales (Harris-Lingoes and Si subscales); (4) Content Scales; (5) Content Component Scales; (6) Supplemental Scales; and (7) PSY-5 Scales (Personality Psychopathology Five Scales). For each scale, the abbreviation, descriptive label, mean score, and standard deviation (SD) are reported.

MMPI-2 Scales		Description of the scales (Fondamenti per l'interpretazione del MMPI-2 e del MMPI-A di James N. Butcher, Carolyn L. Williams, Seconda edizione, Giunti Psychometrics, 2008)	UC patients (Mean T score ±SD)	CD patients (Mean T score ±SD)
VALIDITY SCALES	**CNS ("Cannot Say")**	*The total number of items to which the subject did not respond. This is a measure of the validity of the test that provides the operator with information regarding the desire to collaborate in the psychological evaluation.*	1.03 ± 2.16	1.58 ± 4.30
	**F-K (F minus K)**	*It is used to evaluate the exaggeration of symptoms by comparing the subject's performance on the F scale with the performance on the K scale. It measures a defensive attitude.*	-2.62 ± 9.35	-5.03 ± 10.53
	**Fb (F Back)**	*To identify possible deviant or randomly given answers in the last part of the test. Some subjects, in fact, may change their response attitude to the items as they complete the test and begin to respond in a non-selective or random manner.*	60.97 ± 13.83	57.56 ± 13.15
	**TRIN (True Response Inconsistency)**	*Pairs of items with opposite content to evaluate the tendency that some people have to respond affirmatively or negatively indipendently of the content of items*	59.44 ± 6.81	59.44 ± 6.81
	**VRIN (Variable Response Inconsistency)**	*The calculation of the scale score is done by adding the number of inconsistent answers. Assess for an inconsistent or confused response style.*	56.00 ± 7.91	53.83 ± 9.66
	**Fp (F-psychopathology)**	*It provides a valuable assessment of the authenticity of the subject's symptoms. It assesses the extent to which a person undergoing the exam reports more psychological symptoms than psychiatric patients in outpatient therapy.*	53.14 ± 9.77	50.53 ± 8.38
	**S (Superlative Self-Presentation)**	*Useful for assessing the tendency to recognize positive attributes, high moral values and denial of adaptation problems*	44.48 ± 11.18	44.19 ± 10.36
	**L ("Lie" / Uncommon Virtues)**	*It assesses the tendency of some people to falsify their responses in order to present an overly virtuous image of themselves. The scale identify the tendency to provide a socially desirable image and to deny defects or weakness admitted by the majority of population*	52.52 ± 9.25	53.11 ± 9.78
	**F (Infrequency)**	*An excellent indicator of a person's collaborative mindset and capacity to share meaningful information about themselves. This scale is based on the concept that persons who are seeking to demonstrate psychological adjustment difficulties that they do not have will respond in an excessive manner, responding to all symptoms selected from problem areas that are too broad and incompatible with each other.*	58.76 ± 11.44	57.56 ± 11.99
	**K (Defensiveness)**	*Designed as a measure to assess the defensive attitude toward the test. It is used to mitigate the tendency to deny difficulties.*	43.31 ± 9.37	47.61 ± 10.63
CLINICAL SCALES	**Hs (Hypochondriasis)**	*Abnormal, psychoneurotic concern over one's health. General malaise, pain, weakness, exhaustion, and illness, stomach difficulties, difficulty breathing, poor visual perception and other sensory disorders, cough, difficulty sleeping, dizziness, and body numbness are all mentioned.*	66.66 ± 11.23	67.67 ± 11.86
	**D (Depression)**	*The scale reveals a clinical picture of a usually "negative" psychic structure: the subject reports feeling low in spirits, without hope for the future, dissatisfied with life, and having a gloomy mood. The items' content is obviously associated with depression, low self-esteem, loss of interest, and feelings of indifference.*	63.21 ± 14.88	63.69 ± 14.60

	**Hy (Hysteria)**	*Somatic complaints, denial of psychiatric disorders, extroversion, or comfort in social relationships are all described in the items.*	61.17 ± 14.47	63.47 ± 12.30
	**Pd (Psychopathic Deviate)**	*The overall score is closely connected with exterior actions that indicate aggressive, manipulative, and impulsive behavioral or family difficulties.*	59.28 ± 12.91	59.42 ± 13.67
	**MF (Masculinity/Femininity)**	*The items cover interests and profession choices, with a small number reflecting psychological issues or symptoms. Occupational options are either stereotypically feminine (for example, librarian, nurse, flower artist) or masculine (for example, soldier, sports journalist, forest ranger).*	51.72 ± 8.57	50.72 ± 9.33
	**Pa (Paranoia)**	*It assesses behavioral features associated with paranoid-type disorders, such as suspicion, distrust, fixed thoughts, excessive interpersonal sensitivity, rigid thinking, and critical attitude.*	58.00 ± 12.17	56.08 ± 11.48
	**Pt (Psychasthenia)**	*It was originally designed to assess a psychological disorder (psychasthenia), which we now refer to as anxiety disorders with obsessive-compulsive features. It includes items that evaluate anxiety or overall maladjustment.*	61.38 ± 12.25	57.61 ± 13.75
	**Sc (Schizophrenia)**	*High scores on this scale reflect a wide range of diagnostic possibilities, as significant elevations can occur in subjects with a variety of disorders, including schizophrenics, chronic psychiatric patients with emotional disorders, people with organic brain disorders, severely disturbed personalities, normal subjects with serious sensory system damage, non-conformists, rebels, and "counterculture" (for example, hippies in the 1960s).*	60.69 ± 10.69	57.31 ± 11.98
	**Ma (Hypomania)**	*It assesses manic or hypomanic behavior, which is characterized by feelings of euphoria, aggression, and hyperactivity.*	55.76 ± 14.25	49.78 ± 8.89
	**Si (Social Introversion)**	*The items' content addresses social discomfort, inferiority, poor sociability, interpersonal sensitivity, distrust.*	57.93 ± 11.17	57.17 ± 13.74
**CLINICAL SUBSCALES**	**D_1**	**Subjective Depression**	61.37 ± 14.48	60.66 ± 15.07
	**D_2**	**Psychomotor Retardation**	54.56 ± 12.24	56.14 ± 13.19
	**D_3**	**Physical Malfunctioning**	65.05 ± 13.68	66.00 ± 12.84
	**D_4**	**Mental Dullness**	58.48 ± 14.72	56.96 ± 15.45
	**D_5**	**Brooding**	58.06 ± 11.66	54.17 ± 14.45
	**Hy_1**	**Denial of Social Anxiety**	46.60 ± 9.41	46.87 ± 12.42
	**Hy_2**	**Need for Affection**	46.23 ± 8.95	49.10 ± 10.73
	**Hy_3**	**Lassitude Malaise**	64.17 ± 12.63	62.73 ± 13.35
	**Hy_4**	**Somatic Complaints**	62.66 ± 12.99	64.00 ± 12.16
	**Hy_5**	**Inhibition of Aggression**	46.42 ± 9.04	49.44 ± 10.68
	**Pd_1**	**Familial Discord**	54.74 ± 12.19	55.09 ± 12.49
	**Pd_2**	**Authority Problems**	50.84 ± 10.47	51.82 ± 9.66
	**Pd_3**	**Social Imperturbability**	47.73 ± 8.47	48.20 ± 12.59
	**Pd_4**	**Social Alienation**	59.46 ± 12.72	59.51 ± 13.61
	**Pd_5**	**Self-Alienation**	59.81 ± 11.15	57.93 ± 13.94
	**Pa_1**	**Persecutory Ideas**	60.47 ± 12.23	57.18 ± 13.58
	**Pa_2**	**Poignancy**	59.37 ± 11.68	56.24 ± 12.73
	**Pa_3**	**Naivete**	45.64 ± 9.24	48.55 ± 9.83

	**Sc$_1$**	**Social Alienation**	59.55 ± 12.45	56.54 ± 17.62
	**Sc$_2$**	**Emotional Alienation**	54.96 ± 13.51	54.05 ± 12.89
	**Sc$_3$**	**Lack of Ego Mastery-Cognitive**	60.64 ± 14.68	58.84 ± 14.03
	**Sc$_4$**	**Lack of Ego Mastery-Conative**	57.90 ± 15.68	55.20 ± 13.78
	**Sc$_5$**	**Lack of Ego Mastery-Defective Inhibition**	59.82 ± 12.60	51.78 ± 10.26
	**Sc$_6$**	**Bizarre Sensory Experiences**	63.53 ± 12.18	59.57 ± 13.19
	**Ma$_1$**	**Amorality**	52.77 ± 8.30	50.34 ± 8.30
	**Ma$_2$**	**Psychomotor Acceleration**	54.72 ± 12.46	50.72 ± 11.00
	**Ma$_3$**	**Imperturbability**	45.24 ± 10.09	45.49 ± 10.89
	**Ma$_4$**	**Ego Inflation**	54.05 ± 11.66	50.20 ± 11.57
	**Si$_1$**	**Shyness/Slef-Consciousness**	52.46 ± 9.43	53.07 ± 11.41
	**Si$_2$**	**Social Avoidance**	54.43 ± 10.09	55.52 ± 9.50
	**Si$_3$**	**Self/Other Alienation**	59.94 ± 11.59	57.49 ± 11.94
**CONTENT SCALES**	**ANX (Anxiety)**	*Detects widespread anxiety symptoms (tension, physical issues such as tachycardia and shortness of breath, sleep difficulties, excessive worrying, and concentration difficulties).*	62.97 ± 12.40	59.31 ± 14.24
	**FRS (Fears)**	*Detects the presence of specific fears or phobias associated with various circumstances or things (blood, heights, money, animals such as snakes, mice, or spiders, leaving the house, fire, storms, and natural disasters, water, darkness, being in confined spaces, and dirt).*	58.45 ± 9.05	53.75 ± 8.78
	**OBS (Obsessiveness)**	*Detects ruminative and compulsive thinking cognitive processes.*	58.17 ± 12.25	54.89 ± 12.86
	**DEP (Depression)**	*Determine the presence of symptomatic depression (depressive-type thoughts, emotions of melancholy and uncertainty about one's future, loss of interest in one's life, increased emotional lability, feelings of emptiness, suicidal thoughts, wish for death).*	59.76 ± 19.97	57.44 ± 14.57
	**HEA (Health Concerns)**	*Reports medical symptoms and complaints. Contains items that refer to a variety of physical symptoms that affect different parts of the body, such as gastrointestinal symptoms (constipation, nausea and vomiting, stomach upset), neurological problems (seizures, dizziness, fainting, paralysis), sensory problems (poor hearing or vision), cardiovascular symptoms (chest or heart pain), dermatological problems, pain (headache, neck pain), and breathing difficulties (cough, hay fever, asthma).*	66.86 ± 10.03	66.75 ± 10.67
	**BIZ (Bizarre Mentation)**	*Detects severe symptoms of thought disorders (auditory, visual, or olfactory hallucinations, weird and peculiar thoughts, paranoid ideation, a sense of having a specific task to complete, or exceptional powers).*	56.79 ± 9.83	55.08 ± 9.71
	**ANG (Anger)**	*Examine the loss of control that occurs when rage is expressed.*	56.41 ± 12.07	54.58 ± 13.60
	**CYN (Cynicism)**	*This scale assesses cynical ideas and misanthropic attitudes.*	57.69 ± 12.84	56.08 ± 11.23
	**ASP (Antisocial Practices)**	*It assesses antisocial personality traits (misanthropic sentiments similar to those indicated by high CYN scale scores).*	54.28 ± 12.03	50.61 ± 9.70
	**TPA (Type A Behaviouor)**	*They identify an aggressive, hostile, and competitive personality style.*	56.14 ± 11.90	53.75 ± 11.25
	**LSE (Low Self Esteem)**	*It identifies negative self-perception (a tendency to represent oneself negatively and have a low opinion of oneself).*	55.34 ± 10.66	55.42 ± 13.80

	**SOD (Social Discomfort)**	*Assess social discomfort (the tendency to feel uncomfortable when around others and the urge to be alone).*	57.17 ± 12.81	57.72 ± 13.41
	**FAM (Family Problems)**	*It focuses on family relationship issues (significant family discomfort, family that lacks affection, quarrelsome and unpleasant, hostility towards members of their family).*	57.10 ± 9.85	55.31 ± 13.11
	**WRK (Work Interference)**	*Identifies difficulties and negative attitudes related to work or goal pursuit.*	59.38 ± 13.05	58.39 ± 15.27
	**TRT (Negative Treatment Indicators)**	*Focuses on attitudes or difficulties experienced when seeking treatment or changing behavior (e.g., negative attitudes toward doctors and mental health therapies).*	58.34 ± 11.87	56.75 ± 14.31
**CONTENT COMPONENT SCALES**	**FRS1**	**Generalized Fearfulness**	58.28 ± 10.09	54.36 ± 9.81
	**FRS2**	**Multiple Fears**	55.14 ± 10.10	51.17 ± 7.79
	**DEP1**	**Lack of Drive**	59.24 ± 13.55	56.25 ± 14.22
	**DEP2**	**Dysphoria**	58.14 ± 11.07	55.06 ± 13.93
	**DEP3**	**Self-Depreciation**	56.07 ± 12.00	56.17 ± 14.29
	**DEP4**	**Suicidal Ideation**	57.03 ± 10.70	57.28 ± 8.55
	**HEA1**	**Gastrointestinal Symptoms**	58.34 ± 10.44	59.94 ± 11.42
	**HEA2**	**Neurological Symptoms**	64.21 ± 8.99	62.42 ± 11.63
	**HEA3**	**General Health Concerns**	67.62 ± 11.11	68.00 ± 11.79
	**BIZ1**	**Psychotic Symptomatology**	53.24 ± 10.64	52.17 ± 9.77
	**BIZ2**	**Schizotypal Characteristics**	57.97 ± 10.40	54.28 ± 11.97
	**ANG1**	**Explosive Behavior**	53.24 ± 10.78	53.75 ± 13.69
	**ANG2**	**Irritability**	58.38 ± 13.48	54.39 ± 12.92
	**CYN1**	**Misanthropic Beliefs**	56.52 ± 12.80	55.28 ± 10.79
	**CYN2**	**Interpersonal Suspiciousness**	58.52 ± 11.33	56.36 ± 9.40
	**ASP1**	**Antisocial Attitudes**	54.00 ± 11.26	50.08 ± 9.47
	**ASP2**	**Antisocial Behavior**	52.86 ± 11.21	52.50 ± 8.65
	**TPA1**	**Impatience**	58.52 ± 11.95	53.42 ± 10.91
	**TPA2**	**Competitive Drive**	53.41 ± 10.54	53.11 ± 10.72
	**LSE1**	**Self-Doubt**	55.28 ± 10.91	54.78 ± 12.44
	**LSE2**	**Submissiveness**	52.59 ± 10.05	52.81 ± 11.45
	**SOD1**	**Introversion**	58.10 ± 13.44	59.08 ± 12.53
	**SOD2**	**Shyness**	53.14 ± 8.85	52.92 ± 11.38
	**FAM1**	**Family Discord**	55.07 ± 10.01	53.72 ± 11.40
	**FAM2**	**Familial Alienation**	53.41 ± 8.39	53.00 ± 10.37
	**TRT1**	**Low Motivation**	56.93 ± 11.62	56.92 ± 13.32
	**TRT2**	**Inability to Disclose**	57.62 ± 9.70	53.67 ± 11.36
**SUPPLEMENTAL SCALES**	**A (Anxiety)**	*Items that assess general maladjustment or emotional disturbance are included.*	59.26 ± 11.68	56.18 ± 13.51
	**R (Repression)**	*High scores indicate a lack of desire to express one's concerns in an attempt to suppress knowledge of one's difficulties and hence insight, as well as difficulty in connecting with one's emotional environment.*	51.20 ± 11.68	54.23 ± 10.67

	**Es (Ego Strength)**	*To evaluate the individual's personal resources that allow not to experience emotional distress or maladjustment*	35.27 ± 12.06	36.69 ± 12.10
	**Do (Dominance)**	*To assess personality traits related to leadership. It assesses personality traits such as comfort in social situations, self-confidence, strong opinions, perseverance in commitments, and the capacity to concentrate.*	42.31 ± 9.95	42.67 ± 9.25
	**Re (Social Responsibility)**	*It was created to assess a person's sense of responsibility to others.*	46.97 ± 11.42	49.67 ± 11.46
	**Mt (College Maladjustment)**	*The scale was created to distinguish emotionally well-adjusted college students from those with more serious psychological issues. There are three necessary components: 1) low self-esteem; 2) loss of vitality; 3) cynicism/discontent. Students with high scores are less successful, gloomy, prone to procrastination, worried, and so forth.*	61.34 ± 11.94	59.67 ± 14.46
	**PK (Post-Traumatic Stress Disorder – Keane)**	*It was designed to screen for PTSD symptoms in war veterans (anxiety, depression, emotional discomfort, sleep difficulties, unsettling thoughts, and emotions of being misunderstood or mistreated).*	61.97 ± 13.35	58.89 ± 14.69
	**MDS (Marital Distress)**	*Contains items relevant to marital or relationship troubles.*	55.55 ± 10.02	56.64 ± 12.76
	**Ho (Hostility)**	*It was created to discover people who can work well in groups, establish relationships with others, and keep group morale high.*	58.52 ± 11.63	55.86 ± 11.42
	**O-H (Overcontrolled Hostility)**	*Subjects with high scores exhibit proper behavior but may occasionally express extreme rage. Moreover, this scale indicates a lack of desire to communicate aggression verbally.*	46.00 ± 8.35	50.83 ± 10.11
	**MAC-R (MacAndrew-Revised)**	*It was created to evaluate alcohol consumption issues in therapeutic settings.*	50.45 ± 9.37	48.61 ± 12.80
	**AAS (Addiction Admission)**	*It was created as a measure of openness to admitting the presence of alcohol or drug issues.*	53.38 ± 8.95	57.08 ± 11.33
	**APS (Addiction Potential)**	*It was created to assess the personality traits that contribute to the development of addiction diseases.*	47.62 ± 9.58	46.28 ± 11.65
	**GM (Gender Role – Masculine)**	*The key elements are as follows: 1) denial of fears and anxieties; 2) pleasure in generally male activities; 3) pleasure in typically female activities; 4) denial of excessive emotionality; and 5) emotions of independence and security. In the absence of psychopathology, high scores indicate well-being and excellent functioning. Subjects with these elevations appear to enjoy masculine activities and have little fear, appearing self-assured and making judgments easily.*	42.86 ± 8.50	42.81 ± 8.67
	**GF (Gender Role – Feminine)**	*The key elements are as follows: 1) rejection of antisocial behavior; 2) delight in generally feminine activities; 3) aversion to typically male activities; 4) acknowledgment of even excessive emotional features; and 5) expressions of happiness with one's gender. Female attitudes and delight in traditionally feminine activities are associated with high scores. Subjects with high scores have good interpersonal relationships, avoid disputes, and are socially responsible.*	47.21 ± 9.99	46.89 ± 8.92
**PSY-5 (Personality Psychopathology Five) SCALES**	**AGGR (Aggressiveness)**	*It assesses the likelihood of outrageous and instrumental aggressiveness. The scale also assesses cognitive processes that encourage or prevent aggressiveness, such as grandiosity vs. egalitarianism. Power and dominance over others are also important to the concept.*	52.62 ± 10.69	51.56 ± 10.58
	**PSYC (Psychoticism)**	*Contact with reality or skewed ideas of the social world and objects are measured. Items in this level relate to anomalous sensory and perceptual experiences, as well as a broken connection with reality. There are a lot of factors on this scale that are related to suspicion. They generally reflect unusual beliefs and attitudes.*	57.24 ± 11.46	53.58 ± 10.57

	**DISC (Disconstraint)**	*It assesses risk aversion, the need for planned and order rather than impulsive behavior, and the presence of translational morality. The items measure antisociality as the difficult to control impulses and delay gratifications*	46.41 ± 11.33	48.28 ± 9.57
	**NEGE (Negative Emotionality/Neuroticism)**	*It assesses the affective tendency to negative feelings, including anxiety, uneasiness, and guilt, all of which cause internal misery.*	55.93 ± 10.80	54.31 ± 13.09
	**INTR (Introversion/Low Positive Emotionality)**	*It assesses the affective disposition, which is associated with difficulty experiencing happy moods, a propensity to avoid social situations, and a lack of enthusiasm in pursuing goals and engaging in activities.*	58.07 ± 15.00	58.36 ± 13.85
